# Green Extraction of Antioxidant Polyphenols from Green Tea (*Camellia sinensis*)

**DOI:** 10.3390/antiox9090785

**Published:** 2020-08-25

**Authors:** Qiong Luo, Jia-Rong Zhang, Hua-Bin Li, Ding-Tao Wu, Fang Geng, Harold Corke, Xin-Lin Wei, Ren-You Gan

**Affiliations:** 1 Department of Food Science & Technology, School of Agriculture and Biology, Shanghai Jiao Tong University, Shanghai 200240, China; luoqiong@sjtu.edu.cn (Q.L.); zhangjiarong@sjtu.edu.cn (J.-R.Z.); 2 Guangdong Provincial Key Laboratory of Food, Nutrition and Health, Department of Nutrition, School of Public Health, Sun Yat-sen University, Guangzhou 510080, China; lihuabin@mail.sysu.edu.cn; 3 Institute of Food Processing and Safety, College of Food Science, Sichuan Agricultural University, Ya’an 625014, China; DT_Wu@sicau.edu.cn; 4 Key Laboratory of Coarse Cereal Processing (Ministry of Agriculture and Rural Affairs), College of Pharmacy and Biological Engineering, Chengdu University, No. 2025 Chengluo Avenue, Chengdu 610106, China; gengfang@cdu.edu.cn; 5 Biotechnology and Food Engineering Program, Guangdong Technion–Israel Institute of Technology, Shantou 515063, China; harold.corke@gtiit.edu.cn; 6 Faculty of Biotechnology and Food Engineering, Technion–Israel Institute of Technology, Haifa 3200003, Israel; 7 Research Center for Plants and Human Health, Institute of Urban Agriculture, Chinese Academy of Agricultural Sciences (CAAS), Chengdu 600103, China

**Keywords:** natural deep eutectic solvents, green tea, catechins, ultrasonic, response surface methodology

## Abstract

In this study, the feasibility of improving the extraction yield of green tea antioxidant polyphenols by the combination of ultrasound-assisted extraction (UAE) and deep eutectic solvents (DESs) was investigated. Choline chloride (ChCl)-glycerol was selected as the best DES among 12 ChCl-based DESs to extract tea antioxidant polyphenols. Subsequently, the influences of extraction parameters on total phenolic content (TPC) values were investigated, and liquid/solid ratio, ultrasonic power, and ultrasonic time were optimized based on the response surface methodology. The optimal extraction conditions were a liquid to solid ratio of 36:1 (mL/g), ultrasonic power of 461.5 W, and ultrasonic time of 21 min, with the highest TPC value of 243 ± 7 mg gallic acid equivalent (mg GAE)/g dry weight (DW), which was 13% higher than that before optimization. In addition, under the optimal extraction conditions, tea polyphenolic extract exhibited higher antioxidant activity compared with conventional extraction methods. Four major catechins in the green tea extracts, including (−)-epicatechin (EC), (−)-epigallocatechin (EGC), (−)-epicatechin gallate (ECG) and (−)-epigallocatechin gallate (EGCG) were identified and quantified by high-performance liquid chromatography. In addition, scanning electron microscopy (SEM) analysis revealed that UAE-DES effectively disrupted the green tea leaf cells, thereby improving tea polyphenol yield. In summary, UAE-DES is an ideal green extraction method for the extraction of tea antioxidant polyphenols.

## 1. Introduction

Currently, the tea plant (*Camellia sinensis*) is cultivated in many countries, such as China and Japan, and tea has become a universally consumed beverage around the world [1]. Green tea has the simplest manufacturing process but the most excellent benefits, and tea antioxidant polyphenols (TPs) constitute the majority of advantageous components in green tea [2]. Catechins are the principal components of tea antioxidant polyphenols, which mainly include two non-ester catechins, (−)-epicatechin (EC) and (−)-epigallocatechin (EGC), and two ester catechins, (−)-epicatechin gallate (ECG) and (−)-epigallocatechin gallate (EGCG) [3,4]. These catechins show potential health benefits, such as anticancer, anti-obesity, antibacterial, antioxidant, and antiviral effects [5,6,7,8]. Nowadays, catechins have been widely applied in the pharmaceutical and food industry. Therefore, it is of great importance to establish an eco-friendly and efficient extraction method for extracting antioxidant polyphenols in green tea.

To date, conventional organic solvents, such as ethanol, acetone, and chloroform, remain the most commonly used solvents to extract bioactive compounds from plant materials [9,10]. However, some organic solvents have several distinct disadvantages, such as high cost, high level of residue, and toxicity [10,11]. Therefore, it is of great necessity to develop new environmentally friendly “green” media to meet the requirements of sustainable development. Recently, the concepts of the eco-sustainability and green economy have led to increased concern being paid to deep eutectic solvents (DESs), new solvents that are emerging as a greener and promising substitute for conventional organic solvents [12,13,14,15,16]. DESs fully meet the principles of green chemistry and are generally recognized as safe (GRAS) [17]. DESs possess some distinct advantages compared to conventional solvents, such as favorable thermal stability, high biodegradability, easy preparation, low cost, and toxicity [18,19,20]. Generally, DESs are prepared by the self-association of hydrogen bond donors (HBD) and hydrogen bond acceptors (HBA). In the preparation of DESs, ChCl is the most widely used HBA, and ChCl-based DESs have been frequently used in various research fields, including the extraction of bioactive compounds from plant materials [21,22,23,24]. Another principle of green extraction is reduced energy consumption by innovative technologies, such as ultrasound [25]. DES-based ultrasound-assisted extraction (UAE) can not only greatly reduce the consumption of solvents, energy and labor, but also can destroy the structure of plant cell walls by acoustic cavitation, and then enhance the yield of bio-active constituents [26,27].

In this study, we developed a rapid and efficient method to extract antioxidant polyphenols from green tea by combining the ultrasonic technique with DESs (UAE-DES), which was rarely reported previously. Among the 12 prepared choline chloride (ChCl)-based DESs, the ChCl-glycerol was finally screened as the most suitable candidate solvent for extracting antioxidant polyphenols from green tea, and the extraction conditions, including liquid to solid, ultrasonic power, and ultrasonic time, were further optimized by the response surface methodology (RSM). Under the optimal conditions, it was observed that the total phenolic content (TPC), the total amount of four major catechins, and antioxidant activity of green tea extract obtained by UAE-DES were all higher than those of the extracts obtained by conventional methods (UAE-ethanol extraction, ethanol extraction, and hot water extraction). In addition, scanning electron microscopy (SEM) analysis revealed that UAE-DES effectively disrupted the green tea leaf cells, thereby improving tea polyphenols yield, indicating a high efficiency of the new method.

## 2. Materials and Methods

### 2.1. Chemicals and Reagents

All the solvents or reagents used were of HPLC or analytical grade. Ethanol, FeCl_3_·6H_2_O, HCl, K_2_S_2_O_8,_ and acetic acid were purchased from Titan Scientific Co., Ltd. (Shanghai, China). Gallic acid was purchased from Energy-Chemical Co., Ltd. (Shanghai, China). ChCl and xylitol were obtained from Adamas Co., Ltd. (Shanghai, China). Na_2_CO_3_ was purchased from Sinopharm Chemical Reagent (Shanghai, China). D-sorbitol, glucose, maleic acid, malonic acid, malic acid, sucrose, and Folin–Ciocalteu’s phenol reagent were all obtained from Macklin Biochemical Co., Ltd. (Shanghai, China). Citric acid, ethylene glycol (EG), and 1,2-propanediol (PD) were purchased from TCI Shanghai (Shanghai, China). Lactic acid and sodium acetate were purchased from Shanghai Lingfeng Chemical Reagent Co., Ltd. (Shanghai, China). Glycerol was purchased from MP Biomedicals Co., Ltd. (Shanghai, China). 2, 4, 6-Tri(2-pyridyl)-s-triazine (TPTZ), [2,2′-azino-bis (3-ethylbenzothiazoline-6-sulfonic acid) diammonium salt (ABTS), and 1,1-diphenyl-2-picrylhydrazyl (DPPH) were purchased from Sigma Aldrich (St. Louis, MO, USA). Commercial standards of catechins [(−)-EC, (−)-EGC, (−)-ECG, and (−)-EGCG, purity ≥ 98%] were purchased from Chengdu RefMedic Biotech Co., Ltd. (Chengdu, China).

### 2.2. Sample Preparation

The selenium-enriched green tea (Enshi Yulu, 1000 g) purchased from Enshi Selenium Impression Agricultural Development Co., Ltd. (Hubei, China), was ground into a fine powder using a tube mill (IKA Tube Mill 100 control) and stored at 4 °C and used within one month.

### 2.3. Preparation of DESs

As shown in Table 1, the two components (ChCl and HBD) of DESs were first mixed with a certain molar ratio (ChCl/HBD). After the addition of 20% (*m/v*) deionized water, the mixture was then heated at 60 °C under gentle stirring until a homogeneous solution was formed. This homogeneous solution was DES, which was finally stored at room temperature.

### 2.4. Extraction of Tea Polyphenols

The initial weight of green tea powder (0.50 g) was put into a 50 mL centrifuge tube, and was then mixed with 10 mL DES solution. The UAE process was conducted by using an ultrasonic processor (JY92-IIN, Ningbo Scientz Biotechnology Co., Ltd. China), and the initial ultrasonication conditions were as follows: an ultrasonic probe with a diameter of 6 mm; ultrasonic power of 325 W; ultrasonic time of 10 min. During the UAE process, the sample was always kept on ice in order to avoid the effect of high temperature on degrading tea polyphenols. All experiments were carried out in triplicate.

For UAE-DES or UAE with ethanol extraction, the green tea powder (0.50 g) and DES or ethanol (18 mL) were mixed in a 50 mL centrifuge tube with a certain liquid/solid ratio. After vortexing, the mixture was immediately treated using the ultrasonic processor.

For ethanol extraction, the green tea powder (0.50 g) and ethanol (18 mL) were mixed in a 50 mL centrifuge tube with a certain liquid/solid ratio, and the extraction of green tea polyphenols was carried out at room temperature (25 °C) shaking for 24 h.

For hot water extraction, the green tea powder (0.50 g) and 85 °C water (18 mL) were mixed in a 50 mL centrifuge tube with a certain liquid/solid ratio, and the extraction was carried out at room temperature (25 °C) for a certain time.

After extraction, the crude extract was centrifuged at 12,000× *g* for 10 min. The finally recovered supernatant was used as the sample for the determination of TPC and antioxidant activity, as well as HPLC analysis. The treated green tea leaf powder was freeze-dried for SEM analysis.

### 2.5. Determination of Total Phenolic Content (TPC)

The TPC was determined using the Folin–Ciocalteu method as reported with minor modifications [28]. Briefly, the Folin–Ciocalteu solution agent (2.5 mL) was added to the properly diluted sample (500 µL) and incubated for 4 min, and then 2 mL Na_2_CO_3_ solution (75 g/L) was added to the mixture and the mixture was incubated for 2 h in the dark. The absorbance of the mixture was measured at 760 nm using a UV-visible spectrophotometer (UV1800, Jinghua Instrument Co., Ltd., Shanghai, China). Gallic acid was used as a standard and the results were expressed as milligrams of gallic acid equivalents (mg GAE)/g dry weight (DW) of samples. All experiments were carried out in triplicate.

### 2.6. Determination of Antioxidant Activity

The antioxidant activity of green tea extracts was determined using ferric-reducing antioxidant power (FRAP) assay, DPPH free radical scavenging assay, and ABTS free radical scavenging assay as described with some modifications by Tang et al. [29] and Yang et al. [30]. All experiments were carried out in triplicate.

For the FRAP assay, a 100 µL sample or FeSO_4_ standard solution was added into 3.0 mL fresh FRAP reagent (sodium acetate buffer: TPTZ solution: FeCl_3_ solution= 10:1:1, *v/v/v*). The reaction mixture was vortexed and incubated at room temperature (25 °C) in the dark for 4 min, and the absorbance was immediately recorded at 593 nm. The results were expressed as mmol Fe (II)/100 g DW.

For the DPPH assay, a 100 µL sample of Trolox standard solution was added into 3.9 mL of DPPH working solution. The reaction mixture was vortexed and incubated at room temperature (25 °C) in the dark for 2 h, and the absorbance was immediately recorded at 515 nm. The results were expressed as mmol Trolox/100 g DW.

For the ABTS assay, a 100 µL sample of Trolox standard solution was added into 3.9 mL of ABTS solution. The reaction mixture was vortexed and incubated at room temperature (25 °C) for 6 min, and the absorbance was immediately detected at 734 nm. The results were expressed as mmol Trolox/100 g DW.

### 2.7. High Performance Liquid Chromatography (HPLC) Analysis

The green tea extract obtained under the optimal condition of UAE-DES was analyzed by HPLC using an Agilent XDB C18 column (5 µm, 4.6 mm × 250 mm; Agilent Technologies, SantaClara, CA, USA). The mobile phase consists of solvent A (0.1% (*v/v*) formic acid in water) and solvent B (acetonitrile). The analysis was performed by a gradient elution program: 0 min, 5% B (*v/v*); 10 min, 16% B (*v/v*); 15 min, 16% B (*v/v*); 20 min, 30% B (*v/v*); 25 min, 100% B (*v/v*); 28 min, 100% B (*v/v*); 29–35 min, 5% B (*v/v*). The sample injection volume was 5 µL, and the flow rate was set at 1.0 mL/min. The retention time and spectra of phenolic compounds were compared with the standard compounds and were quantified basing on the peak areas. The results were expressed as mg/g DW.

### 2.8. Experimental Design

#### 2.8.1. Single-Factor Experiments

The single-factor experiments were performed to analyze the effects of five factors, including liquid/solid ratio, ChCl/glycerol molar ratio, water content in ChCl-glycerol, ultrasonic power, and ultrasonic time, on TPC values, in order to obtain the major factors and their levels.

#### 2.8.2. Response Surface Methodology

A three-factor-three-level Box–Behnken design (BBD) was applied to optimize the extraction conditions. Based on the single-factor experiments, liquid to solid ratio (X_1_), ultrasonic power (X_2_), and ultrasonic time (X_3_) were chosen as independent variables in BBD and tested in a 17-run experiments (with 5 central point runs). As shown in Table 2, each independent variable was coded at three levels, −1, 0, and +1, for low, medium, and high levels, respectively.

### 2.9. Morphology

The surface structures of treated samples were obtained and compared to that of unprocessed green tea leaves powder using a FEI Sirion 200 field-emission scanning electron microscope (FEI Co., Hillsboro, OR, USA).

### 2.10. Statistical Analysis

All values were expressed as the mean ± standard deviation (SD). Design-Expert 8.0.6 software (Trial version, State-Ease, Minneapolis, MN, USA) was used to analyze the multiple regression and estimate the coefficients of the regression model. The statistical importance of the regression coefficient was evaluated by F-test. One-way analysis of variance (ANOVA) was employed to evaluate the accuracy of the conducted model by estimate *F*-value, *P*-value, coefficient of determination (R^2^), and lack of fit. All statistical analyses were performed using the software SPSS (version 25.0, IBM SPSS Statistics, IBM Corp, Somers, NY, USA).

## 3. Results

### 3.1. Selection of DES

**Figure 1:**
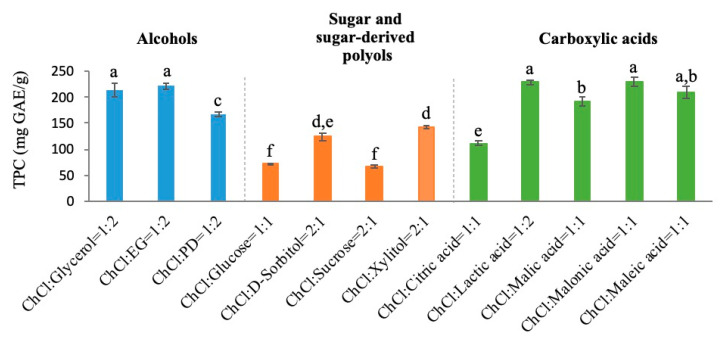
Total phenolic content (TPC) values of the green tea extracts based on 12 selected DESs. Data (a–f) are expressed as means ± SD of *n* = 3 samples.

The screening of extractant for extraction tea polyphenols from green tea leaves is the foremost work that should be done. As shown in Figure 1, the 12 prepared ChCl-based DESs can be further divided into three groups based on the types of HBD, including ChCl-Alcohols DESs, ChCl-Sugar, and ChCl-sugar-derived polyols DESs and ChCl-carboxylic acids DESs.

It was significant that the TPC values in the group of ChCl-sugar, and ChCl-sugar-derived polyols DESs were lower than that in the other two groups. In addition, it could be concluded that five DESs with relatively high TPC value (ChCl-glycerol, ChCl-EG, ChCl-lactic acid, ChCl-malonic acid, and ChCl-maleic acid) were considered as good candidates for green tea polyphenols extraction. However, considering the fact that the corrosiveness or toxicity of lactic acid, malonic acid, maleic acid, and EG, reported in some literature, may causing safety problems, ChCl-glycerol was finally selected as the best DES for further study [31,32,33,34].

### 3.2. Effects of Extraction Parameters on TPC

The effects of extraction parameters on the TPC of green tea extracts were investigated by using single-factor experiments, including the liquid to solid ratio (10:1, 20:1, 30:1, 40:1, and 50:1), ChCl/glycerol molar ratio (1:4, 1:3, 1:2, 1:1, and 2:1), water content in DES-1 (10%, 20%, 30%, 40%, and 50%), ultrasonic power (65, 195, 325, 455, and 585 W), and ultrasonic time (2, 10, 18, 26, and 34 min).

#### 3.2.1. Liquid to Solid Ratio

**Figure 2:**
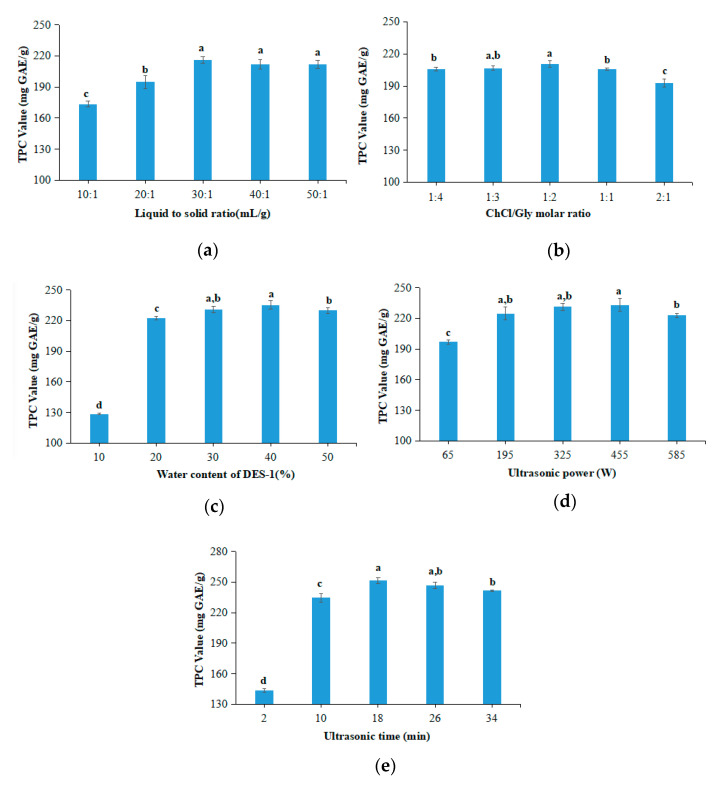
Results of the single-factor experiments. (**a**) Liquid to solid ratio; (**b**) ChCl/glycerol molar ratio; (**c**) water content in ChCl-glycerol; (**d**) ultrasonic power; (**e**) ultrasonic time. Data (a–d) are expressed as means ± SD of *n* = 3 samples.

The liquid to solid ratio was studied in the range of 10:1–50:1. As shown in Figure 2a, an increase in the TPC value was observed with the increase in the liquid to solid ratio from 10:1 to 30:1, and then the TPC value decreased slightly. This is most likely that the level of green tea polyphenols dissolved in ChCl-glycerol was close to saturation at the liquid to solid ratio at 30:1. Therefore, the liquid to solid ratio at 30:1 was selected for the following experiments.

#### 3.2.2. ChCl/Glycerol Molar Ratio

In order to explore the effects of ChCl/glycerol molar ratio on the TPC value of green tea extracts, the extraction was performed using DESs prepared with different ChCl/glycerol molar ratios, from 1:4 to 2:1. As shown in Figure 2b, the TPC value slightly increased with the increase in the ChCl/glycerol molar ratio from 1:4 to 1:2, and then decreased gradually. Therefore, the ChCl/glycerol molar ratio of 1:2 was chosen for the following experiments.

#### 3.2.3. Water Content in the DES-1(ChCl-glycerol)

The extraction was performed using DESs prepared with the addition of a different amount of water (ranging from 10% to 50%), in order to investigate the effects of water content in DES-1 on the TPC value of green tea extracts. As shown in Figure 2c, a significant increase in the TPC value was observed when the water content was changed from 10% to 20%, and then the TPC value increased slowly and reached the maximum at the water content of 40%. The addition of a certain amount of water can reduce the viscosity of DES, which is favorable to enhance the extraction efficiency [20]. However, sequentially increasing the water content in DES-1 caused a significant decrease in the extraction yields. The reason for the decrease was probably because the excessive addition of water destroyed the hydrogen-bonding interactions between ChCl and glycerol, having a de-structuring effect [35]. Therefore, appropriate water content in DES can not only reduce the viscosity of DES, but also keep the number of ChCl-glycerol supramolecular complexes and hydrogen bonds between ChCl and glycerol the same, which may significantly increase the yield of target products. According to our results, the water content of 40% in DES-1 was finally chosen for the following experiments.

#### 3.2.4. Ultrasonic Power

The extraction process was performed using different ultrasonic power ranging from 65 to 585 W, in order to study its effects on the TPC value of green tea extracts. As shown in Figure 2d, the TPC value increased with the increase of ultrasonic power from 65 to 455 W. Samaram et al. reported that the hydrodynamic force was increased with the increase in the ultrasound power, thereby easily disrupting the cell wall and enhancing the yield [36]. However, excessive ultrasound power might cause an increase in bubble numbers in solvents during cavitation, which might reduce the efficiency of the ultrasound energy transmitted into the medium and decrease the yield [37]. Therefore, the ultrasonic power of 455 W was selected for further experiments.

#### 3.2.5. Ultrasonic Time

Different ultrasonic time (from 2 to 34 min) was used to extract polyphenols from green tea, in order to investigate its effects on the TPC value of green tea extracts. As shown in Figure 2e, the highest TPC value was observed at the ultrasonic time of 18 min. The increase in ultrasonic time can facilitate a complete dissolution of bioactive compounds in DES. However, longer ultrasonic time may cause more degradation or decomposition of bioactive compounds in green tea extracts and waste of energy. Therefore, the ultrasonic time of 18 min was selected for the following studies considering both the improvement of extraction efficiency and saving of energy.

### 3.3. Optimization of the Extraction Conditions Using the UAE-DES Method

#### 3.3.1. Model Adequacy

The results of single-factor experiments show that the three factors including liquid to solid ratio, ultrasonic power, and ultrasonic time had greater effects on the extraction efficiency, therefore they were selected for further optimization by RSM. The 17 experimental results for BBD are shown in Table 3. By carrying out multiple regression analysis towards the experimental data, the proposed model was modified as the following quadratic polynomial equation for the variables and response concerning coded levels: Y = 239.16 + 3.46 X_1_ − 0.56 X_2_ + 6.93 X_3_ + 0.65 X_1_X_2_ + 1.48 X_1_X_3_ + 1.63 X_2_X_3_ − 3.66 X_1_^2^ − 4.61 X_2_^2^ − 9.98 X_3_^2^, where Y is the TPC, X_1_ is the liquid to solid ratio, X_2_ is the ultrasonic power, and X_3_ is the ultrasonic time. The results obtained by the software indicate that the predicted maximal TPC value of 241.468 mg GAE/g could be obtained under the optimal conditions as follows: liquid to solid ratio of 35.56: 1, ultrasonic power of 461.10 W and ultrasonic time of 21.13 min. Considering the controllability of the actual operation, the optimal experimental conditions were modified as follows: liquid to solid ratio of 36:1, ultrasonic power of 461.50 W, and ultrasonic time of 21 min.

ANOVA for the second-order response surface model is shown in Table 4. The significance of every coefficient was determined using the *p*-value and checked by the *F*-test. A model term was recognized as significant if its *p*-value was lower than 0.05. Basing on the ANOVA results, the *p*-value was <0.0001, which implied that the regression model was significant. The *F*-value of 37.18 indicated that the model was significant. The linear coefficients (X_1_ and X_3_) and quadratic term coefficients (X_1_^2^, X_2_^2^, and X_3_^2^) were recognized to be significant depending on the principle above. In addition, the coefficient value of determination (*R*^2^) and the adjusted *R*^2^ were 0.9795 and 0.9532, respectively, suggesting a reasonable agreement between the actual and predicted results by the model. Meanwhile, the lack of fit was employed to evaluate the failure of the regression model to express the experimental data which were not included in the range of this regression analysis. The “Lack of fit *F*-value” was 0.25 and the “Lack of fit *p*-value” was more than 0.05, demonstrating that the model explained all the experimental data suitably. Furthermore, a low value of 0.79 of the coefficient of variation (C.V.) implied a good deal of credibility and a high degree of accuracy. In conclusion, the results indicate that the model was sufficient to express the relationship between the variables and response. Consequently, we concluded that the regression model was practically sound.

#### 3.3.2. TPC in Tea Extracts

**Figure 3:**
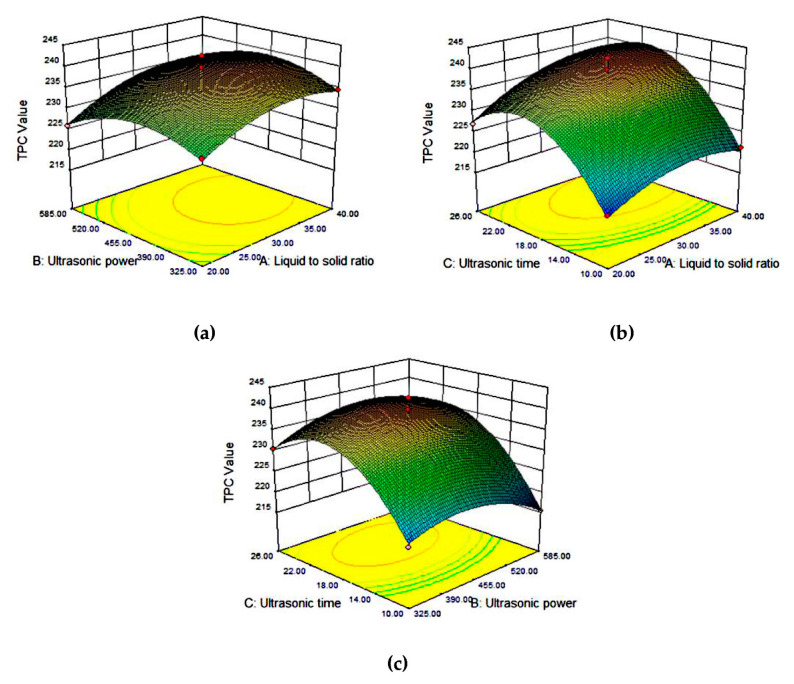
Response surface plots for the interactive effect of (**a**) liquid to solid ratio and ultrasonic power, (**b**) ultrasonic time and liquid to solid ratio, and (**c**) ultrasonic time and ultrasonic power, on the TPC.

As shown in Table 3, the experimental values of TPC ranged between 215 and 243 mg GAE/g. Figure 3a shows the surface response of TPC as a function of ultrasonic power and liquid to solid ratio (at a constant ultrasonic time of 18 min), while Figure 3b shows the TPC response as a function of liquid to solid ratio and ultrasonic time for ultrasonic power at 455 W, and Figure 3c shows the TPC response as a function of ultrasonic power and ultrasonic time (at a constant liquid to solid ratio of 30:1). As seen from Figure 3a, the influence of ultrasonic power was positive until reaching an optimal level and then the TPC decreased. This was probably because the polyphenol ingredients may be degraded under excessively high ultrasonic power. Similar trends were observed with other biomass such as the mung bean coat [38] or the seed coats of red sword bean [39]. The behaviors of the liquid to solid ratio and ultrasonic time were similar to the factor of ultrasonic power. According to the reported literature, a steeper curve in the response surface indicates a more significant influence of the factor [19]. Therefore, it was concluded that the ultrasonic time had the largest effect, but the ultrasonic power had the smallest effect, which is consistent with the conclusion obtained from the regression analysis (Table 4).

#### 3.3.3. Verification of the Predicted TPC Value

In order to verify the adequacy and validity of the regression model, verification experiments under the optimized conditions were carried out. Under the modified optimal conditions, the TPC value was 243 ± 7 mg GAE/g, which was close to the predicted TPC value (241.468 mg GAE/g), and this result demonstrates that the regression model was sufficient for the extraction process.

### 3.4. Comparison of Extraction Methods on TPC and Antioxidant Activity in Green Tea Extracts

As shown in Table 5, under the same extraction time (21 min), the TPC value of the extract obtained by UAE-DES was about 60% and 11% higher than that obtained by hot water extraction and UAE with ethanol, respectively, suggesting a better extraction ability of this DES than ethanol. Moreover, ethanol extraction for 24 h got a similar TPC yield compared to UAE-DES for only 21 min, indicating that the UAE-DES was not only a time-saving but also an efficient approach. Similarly, in many studies, the application of UAE-DES provided a green and highly efficient extraction method for bioactive compounds from plant materials than conventional methods [40,41,42]. For antioxidant activity, UAE-DES also produced higher FRAP, DPPH, and ABTS values than UAE-ethanol, ethanol, and hot water extraction, which may be related to the higher content of polyphenols in the tea extract obtained by UAE-DES, consistent with previous studies that demonstrated that polyphenols were the main contributors to antioxidant activities of teas [28,29].

### 3.5. HPLC Quantification of the Major Catechins in Green Tea Extracts

**Figure 4:**
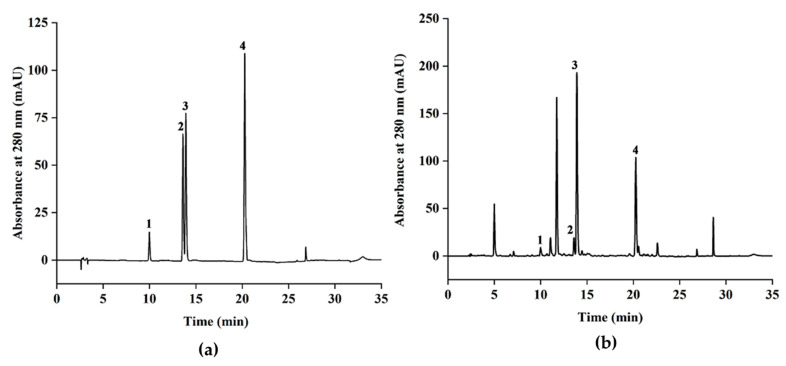
HPLC analysis of the mixture of four commercial standards of catechins (**a**) and green tea extract obtained by UAE-DES (**b**). Peaks 1 to 4 in (**a**) correspond to the commercial standard of (−)-EGC (9.9 min), (−)-EC (13.5 min), (−)-EGCG (13.8 min) and (−)-ECG (20.3 min), respectively.

According to the HPLC analysis (Figure 4), four catechins were identified in the green tea extracts by comparing the retention time and UV spectra with the corresponding commercial standard, including two minor non-ester catechins, (−)-EGC (peak 1) and (−)-EC (peak 2), and two major ester catechins, (−)-EGCG (peak 3) and (−)-ECG (peak 4). These four catechins were then quantified by HPLC using the corresponding commercial standards. In addition, there was a high peak that appeared at about 11.6 min, which was identified as caffeine by comparing its retention time and UV spectrum to the commercial standard of caffeine, a by-product extracted from the green tea. Since caffeine does not belong to polyphenols, it is not further analyzed. As shown in Table 6, it is noteworthy that the contents of all catechins in the green tea extracts obtained by UAE-DES are higher than those obtained by other three conventional methods (UAE-ethanol, ethanol extraction, and hot water extraction), suggesting a higher ability of this UAE-DES method to extract catechins from green tea. The content of (−)-EGCG was the highest, followed by (−)-ECG, (−)-EGC and (−)-EC. These catechins were reported to mainly contribute to the antioxidant properties of the green tea infusions [43,44].

### 3.6. SEM Measurements

**Figure 5:**
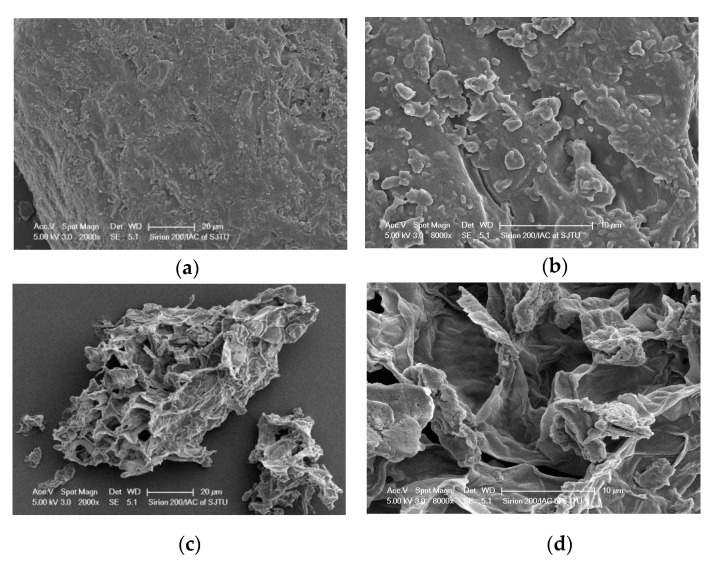
SEM images of the green tea powder before (**a**,**b**) and after ultrasonic treatment (**c**,**d**). Images (**a**,**c**) were taken at 2000× *g* magnification, and (**b**,**d**) at 8000× *g* magnification.

SEM was used to further reveal the potential mechanism of UAE-DES on antioxidant polyphenol extraction in green tea. The magnification at 2000× *g* clearly showed many pores on the tea material surface derived by UAE-DES method (Figure 5c) compared to the sample before extraction (Figure 5a) with an overall smooth surface, which was further supported by the magnification at 8000× *g* (Figure 5b,d). It was clear that the swelling and enlargement of the pores in the materials were observed in the sample of UAE-DES method, suggesting the rupture of cells and the damage of cell structures after UAE-DES treatment, which contributed to easier and efficient penetration of the solvent into the plant material. A similar conclusion was also obtained by others, who reported that ultrasound could induce cavitation phenomena, causing structural changes in cellulose [45,46].

## 4. Conclusions

In this work, an environmentally friendly and powerful UAE-DES method was developed to extract antioxidant polyphenols from green tea. The DES ChCl-glycerol was finally selected as the most suitable DES. Subsequently, single-factor experiments were performed and the main factors influencing the extraction were optimized by RSM. The TPC value was 243 ± 7 mg GAE/g under the following optimized conditions: liquid to solid ratio of 36:1, ultrasonic power of 461.5 W, and ultrasonic time of 21 min. The antioxidant activity and content of four major catechins [(−)-EGC, (−)-EC, (−)-EGCG and (−)-ECG)] of the green tea extracts were also found to be higher than those of the extracts obtained by UAE with ethanol, ethanol extraction, and hot water extraction. In addition, SEM analysis indicated that UAE-DES led to a loose structure and surface erosion of the green tea leaves, which could result in greater penetration of the solvent into the plant material. The results presented in this work indicate that the combination of sustainable green solvents (DESs) and ultrasound-assisted extraction method (UAE) is a good method for the extraction of antioxidant polyphenols from green tea, which has potential applications in the food industry.

## Figures and Tables

**Table 1:** List of the prepared ChCl-based deep eutectic solvents (DESs).

No.	HBD	Molar Ratio
1	Glycerol	1:2
2	EG	1:2
3	PD	1:2
4	Glucose	1:1
5	D-sorbitol	2:1
6	Sucrose	2:1
7	Xylitol	2:1
8	Citric acid	1:1
9	Lactic acid	1:2
10	Malic acid	1:1
11	Malonic acid	1:1
12	Maleic acid	1:1

**Table 2:** Independent variables and their levels used for Box–Behnken design (BBD).

Independent Variables	Coded Levels		
	−1	0	+1
X_1_ (liquid to solid ratio, mL/g)	20	30	40
X_2_ (ultrasonic power, W)	325	455	585
X_3_ (ultrasonic time, min)	10	18	26

**Table 3:** Experimental runs and the results of the response parameters.

Run	X_1_ (Liquid to Solid Ratio, mL/g)	X_2_ (Ultrasonic Power, W)	X_3_ (Ultrasonic Time, min)	Response Y (TPC, mg GAE/g)
1 *	0	0	0	239 ± 2
2	1	0	−1	221 ± 3
3 *	0	0	0	238 ± 1
4 *	0	0	0	237 ± 5
5	−1	−1	0	229 ± 5
6	1	−1	0	234.6 ± 0.6
7	−1	0	1	227 ± 2
8	−1	0	−1	217 ± 1
9 *	0	0	0	243 ± 3
10	0	1	−1	215 ± 2
11	−1	1	0	226 ± 3
12	1	1	0	234 ± 4
13	0	−1	−1	218.9 ± 0.7
14 *	0	0	0	240 ± 2
15	1	0	1	237 ± 4
16	0	−1	1	231 ± 2
17	0	1	1	233.5 ± 0.4

* Five replicates of the central point. Data are expressed as means ± SD of *n* = 3 samples.

**Table 4:** Analysis of variance (ANOVA) of the response surface model.

Source	Sum of Squares	df	Mean Square	*F*-Value	*p*-Value	Significant
Model	1117.99	9	124.22	37.18	<0.0001	significant
X_1_	95.91	1	95.91	28.71	0.0011	
X_2_	2.53	1	2.53	0.76	0.4129	
X_3_	383.64	1	383.64	114.84	<0.0001	
X_1_X_2_	1.69	1	1.69	0.51	0.4999	
X_1_X_3_	8.70	1	8.70	2.61	0.1506	
X_2_X_3_	10.56	1	10.56	3.16	0.1186	
X_1_^2^	56.25	1	56.25	16.84	0.0046	
X_2_^2^	89.29	1	89.29	26.73	0.0013	
X_3_^2^	419.37	1	419.37	125.54	<0.0001	
Residual	23.38	7	3.34			
Lack of fit	3.65	3	1.22	0.25	0.8601	not significant
Pure error	19.73	4	4.93			
Cor total	1141.37	16				
R^2^	0.9795					
Adj. R^2^	0.9532					

**Table 5:** TPC and antioxidant activity of the extracts obtained by different extraction methods.

Extraction Methods	Extraction Time	TPC (mg GAE/g)	FRAP (mmol Fe (II)/100 g DW)	DPPH (mmol Trolox/100 g DW)	ABTS (mmol Trolox/100 g DW)
UAE-DES	21 min	243 ± 7 ^a^	332 ± 9 ^a^	215 ± 6 ^a^	99 ± 3 ^a^
UAE with Ethanol	21 min	219 ± 3 ^b^	285 ± 6 ^c^	195 ± 3 ^c^	77 ± 1 ^c^
Ethanol Extraction	24 h	242 ± 2 ^a^	300 ± 3 ^b^	205 ± 5 ^b^	84 ± 3 ^b^
Hot Water Extraction	21 min	152 ± 2 ^c^	174 ± 5 ^d^	99.0 ± 0.8 ^d^	46 ± 1 ^d^

Different superscript lowercase letters in the same column indicate statistical significance at *p* < 0.05.

**Table 6:** Concentrations of four main catechins in the green tea extracts obtained by different extraction methods.

Compound	Concentration (mg/g DW)			
	EGC	EC	EGCG	ECG
UAE-DES	24.4 ± 0.6 ^a^	8.5 ± 0.4 ^a^	94 ± 2 ^a^	36.2 ± 0.7 ^a^
UAE-Ethanol	19.6 ± 0.5 ^c^	6.8 ± 0.6 ^b^	92.3 ± 0.8 ^a^	35.3 ± 0.7 ^a^
Ethanol Extraction	22 ± 1 ^b^	6.9 ± 0.2 ^b^	92 ± 2 ^a^	35.4 ± 0.4 ^a^
Hot Water Extraction	12.5 ± 0.2 ^d^	4.1 ± 0.1 ^c^	36.8 ± 0.4 ^b^	11.7 ± 0.1 ^b^

Different superscript lowercase letters in the same column indicate statistical significance at *p* < 0.05.
